# Diet Beverage Intake during Lactation and Associations with Infant Outcomes in the Infant Feeding Practices Study II

**DOI:** 10.3390/nu13093154

**Published:** 2021-09-10

**Authors:** Qiushi Huang, Jeanne Murphy, Emily R. Smith, Allison C. Sylvetsky

**Affiliations:** 1 Department of Exercise and Nutrition Sciences, Milken Institute School of Public Health, The George Washington University, 950 New Hampshire Avenue NW, Suite 200, Washington, DC 20052, USA; qhuang@gwmail.gwu.edu (Q.H.); emilysmith@email.gwu.edu (E.R.S.); 2 School of Nursing, The George Washington University, 1919 Pennsylvania Avenue NW, Suite 500, Washington, DC 20006, USA; jeannemurphy@email.gwu.edu; 3 Department of Global Health, Milken Institute School of Public Health, The George Washington University, 950 New Hampshire Avenue NW, Suite 400, Washington, DC 20052, USA

**Keywords:** human milk, breastfeeding, low-calorie sweetener, maternal, infant, nutrition, vomiting

## Abstract

Consumption of diet beverages (DB) containing low-calorie sweeteners (LCS) is widespread in the United States. LCS are ingested by nursing infants upon maternal DB consumption, which may impact infants’ weight and health. This study aims to examine cross-sectional associations between infants’ LCS exposure via maternal DB intake during lactation and infants’ health outcomes. Six hundred and eighty-two mother–infant dyads at three months postpartum, from the Infant Feeding Practices Study II, 2005–2007, were included in the analysis. Maternal DB consumption during lactation was estimated using the serving size and frequency of DB consumption reported on the diet history questionnaire. Infants’ LCS exposure was estimated by multiplying maternal DB consumption and breastfeeding intensity. Infant outcomes included weight, weight-for-age and BMI-for-age z-scores, overweight, and gastrointestinal (GI) symptoms including diarrhea, reflux, and vomiting. Associations between infants’ LCS exposure and continuous and categorical outcomes were examined using linear and logistic regressions adjusting for confounders, respectively. Forty-three percent of lactating women reported DB consumption. While no significant associations were observed between infants’ LCS exposure and BMI-for-age or risk of overweight, infants’ LCS exposure was associated with a 2.78-fold increased risk of vomiting (95% confidence interval 1.05–7.34). Potential adverse effects of LCS exposure on GI symptoms require further study, and null findings on infant weight should be interpreted with caution, given the small sample size. Additional research is needed to inform recommendations for or against DB consumption during lactation.

## 1. Introduction

Reducing added sugar intake, and specifically, consumption of sugar-sweetened beverages, is a key dietary recommendation for prevention of excessive weight gain and cardiometabolic disease, both in the general population and among pregnant and lactating women [1,2]. A common approach to lowering added sugar intake is replacement with low-calorie sweeteners (LCS), which provide sweetness, but do not contain sugar or calories. LCS consumption is widespread among children (25%) [3], adults (41%) [3], and pregnant women (24%) [4] in the United States (US), with diet beverages (DBs) comprising the majority of LCS intake [5]. Meanwhile, the number of consumer products containing LCS has increased four-fold in recent years [5], and this trend is expected to continue with ongoing public health efforts to reduce consumption of added sugars.

LCS including aspartame, sucralose, and acesulfame-potassium are widely found in DBs, are approved by the US Food and Drug Administration (FDA) as food additives, are not believed to be carcinogenic, and are considered safe for human consumption at levels below the acceptable daily intake (ADI) [6]. However, there is growing uncertainty surrounding the role of LCS in the development of diet-related chronic diseases [7], with particularly sparse evidence surrounding health effects of LCS when exposure begins early in life [8]. The few studies that have recently been conducted support the likelihood that maternal LCS consumption during pregnancy and/or lactation may have adverse effects on offspring weight and health [8,9,10,11]. For example, maternal intake of beverages containing LCS during pregnancy was associated with perturbations in gut microbiota and a higher infant body mass index [11]. In rodents, exposure to LCS during pregnancy and/or lactation, even at non-pharmacologic doses, impaired hepatic detoxification pathways and disturbed the gut microbiome composition consistent with patterns observed in metabolic disease [12].

We have previously demonstrated that LCS are present in human milk [13] and are ingested by nursing infants after acute ingestion of a DB [14]. However, the prevalence of DB use among lactating women is presently unknown, and recommendations for or against maternal LCS consumption during lactation are currently lacking. Herein, we describe the prevalence of DB consumption in a nationally-distributed sample of lactating women in the US, and examine associations between infants’ LCS exposure via maternal DB intake during lactation and infants’ health outcomes at three months of age.

## 2. Materials and Methods

In this cross-sectional analysis, we utilized maternal and infant data collected approximately three months postpartum in the Infant Feeding Practices Study II (IFPSII) [15]. The IFPSII was a longitudinal study conducted by the US Centers for Disease Control and Prevention (CDC) and US Food and Drug Administration (FDA) from 2005–2007. Women were followed from late pregnancy through the infant’s first year of life. A sample of 4902 pregnant women, recruited from a nationwide consumer opinion panel of >500,000 households, was determined to be eligible for IFPSII participation. Eligibility criteria included maternal age ≥ 18 years, singleton birth born at ≥ 35 weeks gestation, infant birthweight ≥ 5 pounds (2.3 kg), and absence of a medical condition at birth that would affect the infants’ feeding. Any infant that stayed in intensive care for more than 3 days following birth was excluded.

**Figure 1:**
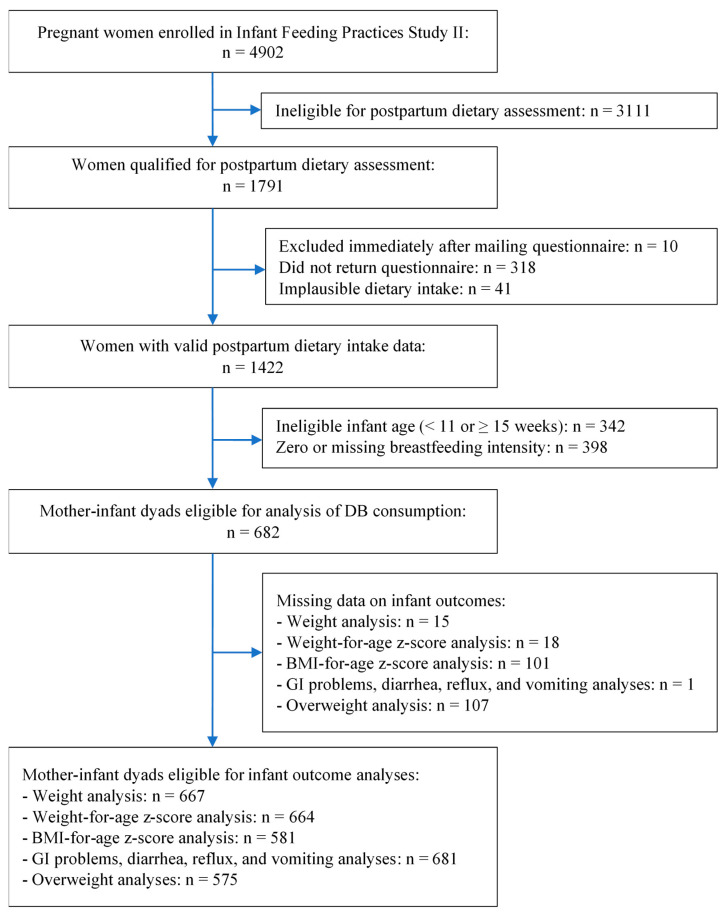
Flow diagram of analytical sample of lactating mother–infant dyads in IFPS II.

Among 1791 lactating women eligible for postpartum dietary assessment, 1422 returned the questionnaire with valid dietary data; 342 women were excluded due to ineligible infant age (infant < 11 or ≥15 weeks old), and an additional 398 women were excluded due to zero or missing breastfeeding intensity. The final analytic sample included 682 lactating women and their infants (hereafter mother–infant dyads). A detailed sample flow chart is presented in Figure 1.

A modified version of the Diet History Questionnaire (DHQ) was used in IFPSII to assess maternal diet by changing the time frame of recall to 1 month and adding specific foods of interest for pregnant women [15]. Maternal soda (diet and regular) consumption was estimated from reported consumption of “soft drinks, soda, or pop” on the postpartum DHQ (0.5 serving = < 12 oz or 1 can/bottle, 1 serving = 12–16 oz or 1 can/bottle, 2 servings = > 16 oz or 1 can/bottle). Frequency of total (diet and regular) soda consumption was converted to number of times per month. Consumption of diet soda was then estimated by the cross product of total maternal soda consumption and the percentage of soda consumed that was reported to be diet or sugar-free (0: almost never or never, 25%: about ¼ of the time, 50%: about ½ of the time, 75%: about ¾ of the time, 100%: almost always or always). Maternal consumption of total and diet fruit drinks was estimated similarly, with serving sizes defined as 0.5 serving (<8 oz or 1 cup), 1 serving (8–16 oz or 1–2 cups), or 2 servings = > 16 oz or 2 cups). Total maternal DB consumption was then calculated as the sum of intakes of diet soda and diet fruit drinks, and further categorized into four mutually exclusive DB intake groups (never, ≤1 serving/week, <1 serving/day, ≥1 serving/day).

For analyses of infant outcomes, infants’ LCS exposure via maternal DB intake was estimated by the cross product of maternal DB consumption and breastfeeding intensity. Breastfeeding intensity was operationalized as the percentage of all milk feeds that were human milk, per mothers’ self-report on the postnatal questionnaire. Infants’ LCS exposure via maternal DB intake was then categorized dichotomously as exposed and non-exposed. For supplemental analyses, infants’ LCS exposure via maternal DB intake, accounting for breastfeeding intensity, was further categorized into four mutually exclusive groups (never, ≤1 serving/week, <1 serving/day, ≥1 serving/day).

Infant weight was reported by the mother on the postnatal questionnaire and converted to kilograms. Weight-for-age z-scores were calculated using the World Health Organization (WHO) Child Growth Reference Standards [16]. Any reported gastrointestinal (GI) symptoms (yes/no) was defined as having had diarrhea, reflux or vomiting during the two weeks prior to completion of the postnatal questionnaire. In a subset of infants for whom data on infant length (reported by the mother) was available (*n* = 581), we also examined BMI-for-age z-score and infant overweight status (yes/no, defined as weight-for-length z-score >97.7th percentile [16]).

We considered the following potential confounders: maternal age (years), race/ethnicity (white, black, Hispanic, other), household income (<$35,000, $35,000–$74,999, ≥$75,000), education level (high school or less, some college, college or above), marital status (yes/no) and gestational diabetes status (yes/no) based on self-reported responses on the demographic and prenatal questionnaires; pre-pregnancy BMI (kg/m^2^) calculated from self-reported height and weight on the prenatal questionnaire; gestational weight gain (GWG, pounds) based on self-reported responses to the neonatal questionnaire; adherence to GWG recommendations (insufficient GWG, in healthy range, excess GWG) determined using pre-pregnancy BMI and GWG in accordance with the Institute of Medicine (IOM) Weight Gain Recommendations for Pregnancy [17]; total maternal energy intake (kcal/day) estimated using dietary intake data from the postpartum DHQ; maternal sugar-sweetened beverage (SSB) intake (servings/month) estimated by subtracting maternal DB consumption from the consumption of all sweetened beverages; infant’s birth weight (pounds) reported by the mother on the birth screener; and exclusive breastfeeding (yes/no) derived from infant’s food intake reported by the mother on the month-3 postnatal questionnaire.

Exclusive breastfeeding and pre-pregnancy weight status were also considered potential effect modifiers in the association between infants’ LCS exposure and infant outcomes. Additionally, because previous research has shown that several foods, including brassica vegetables and garlic, induce GI upset, we also explored maternal ingestion of GI upsetting foods as effect modifiers in the association between infants’ LCS exposure and any reported GI symptoms, using broccoli as a proxy. Intake of broccoli was categorized dichotomously (no intake versus any intake) based on dietary intake data from the postpartum DHQ.

Throughout the IFPSII study period, a series of questionnaires (demographic, diet history, prenatal, birth screener, neonatal, and postnatal) were administered by mail, with response rates ranging from 63–87% [15]. The demographic questionnaire was mailed to all women during study recruitment. The prenatal questionnaire was mailed when the mothers were approximately 7 months pregnant. Mothers completed a short birth screener interview by telephone around the time of infant’s birth. The neonatal questionnaire was completed at approximately 1 month postpartum. The postnatal questionnaires with varying topics were mailed every month between month 2 and month 12, except for month 8. The postpartum DHQ was mailed approximately 3 months postpartum.

### Statistical Analysis

Mean, standard deviation (SD), and frequency of sociodemographic characteristics were compared across maternal DB consumption categories using chi-square and ANOVA tests for categorical and continuous variables, respectively. Associations between infants’ LCS exposure via maternal DB intake with infant weight, weight-for-age, and BMI-for-age z-scores were examined using linear regression adjusting for potential confounders, including maternal age, race/ethnicity, household income, education level, marital status, gestational diabetes status, pre-pregnancy BMI, GWG, total energy intake, SSB intake, infant’s birth weight, and exclusive breastfeeding. Associations between infants’ LCS exposure via maternal DB intake with any reported GI symptoms, diarrhea, reflux, vomiting, and infant overweight were examined using logistic regression adjusting for the same confounders. Potential effect modifications by exclusive breastfeeding and pre-pregnancy weight status were examined by including the interaction term between each variable and infants’ LCS exposure in all models. Potential effect modification by maternal ingestion of broccoli was examined by including the interaction term between intake of broccoli and infants’ LCS exposure in the model for GI symptoms. Mean (SD) and prevalence of infant outcomes by categories of infants’ LCS exposure via maternal DB intake, as well as unadjusted and adjusted mean differences, odds ratios (OR), and 95% confidence intervals (CI) were reported for continuous and categorical outcomes, respectively. Supplemental analyses were performed with infants’ LCS exposure divided into four categories, using the same statistical procedures. Missing data on potential confounders were addressed using median and mode imputation for continuous and categorical variables, respectively. Additionally, a complete case analysis excluding all observations with any missing data was conducted as a sensitivity analysis. Statistical significance was considered present at *p* < 0.05. All analyses were performed using SAS version 9.4 (SAS Institute, Cary, NC, USA).

## 3. Results

Characteristics of the study sample are shown, by maternal DB consumption, in Table 1.

Approximately 43.4% of women reported consuming DBs, and 15.3% reported consumption of at least one serving (8–16 ounces per DHQ used in IFPSII) of DBs per day. Frequency of DB consumption varied by race/ethnicity, with a higher percentage of white women reporting daily DB intake, compared with mothers who self-identified as black, Hispanic, or other. Having gestational diabetes and higher pre-pregnancy BMI was also positively associated with more frequent DB intakes. Daily energy intakes were lower among women who reported DB intake compared to non-consumers, yet no differences in gestational weight gain were observed based on maternal DB consumption.

Mean (±SD) infant body weight (kg), weight-for-age z-score, and BMI-for-age z-score by infants’ LCS exposure are shown in Table 2. There were no statistically significant differences in infants’ weight or z-scores based on LCS exposure before or after adjustment for potential confounders.

Prevalence of any reported GI symptoms, diarrhea, reflux, vomiting, or overweight, by infants’ LCS exposure, are shown in Table 3. Infants exposed to LCS had a significantly higher risk of vomiting compared to those not exposed, after adjusting for potential confounders (adjust OR = 2.78, 95% CI 1.05–7.34). No significant associations were observed for other outcomes. No significant effect modifications by exclusive breastfeeding, pre-pregnancy weight status, or intake of broccoli on the association between infants’ LCS exposure and outcomes were observed, and thus, were excluded from the final models.

In supplemental analyses, compared with infants not exposed to LCS, increased risk of vomiting was observed only among infants with LCS exposure of ≤ 1 serving/week (adjusted OR = 3.49, 95% CI 1.00–12.10). No such increases were observed among infants with higher LCS exposure (<1 serving/day and ≥1 serving/day). No significant differences in the risk of other infant outcomes were observed across the four categories of infants’ LCS exposure (Supplemental Tables S1 and S2).

In the complete case analysis, LCS exposure via maternal DB intake was associated with a significantly higher risk of vomiting after adjusting for potential confounders (adjusted OR = 3.20, 95% CI 1.12–9.13) (Supplemental Table S3). However, compared with infants not exposed to LCS, no differences in the risk of vomiting was observed among infants with LCS exposure of ≤1 serving/week, <1 serving/day or ≥1 serving/day (Supplemental Table S3). No associations between infant LCS exposure and other infant outcomes were observed in the complete case analysis (data not shown).

## 4. Discussion

Our findings confirm that DB consumption is widespread among lactating women in the US. Given that these estimates are based on data collected more than a decade ago, and consistent with trends in DB intake reported in pregnant women [4] and the general US population [3], the current prevalence of DB consumption among lactating women in the US likely far exceeds what is reported herein. Nonetheless, these results provide the first estimates of DB intake among lactating women, which to our knowledge, have not been previously documented in the US or elsewhere. This is critically important given the continued uncertainty about effects of early life LCS exposure on infants’ weight and health [18].

While no statistically significant associations were observed between infants’ LCS exposure via maternal DB intake during lactation and infants’ weight outcomes at three months of age, infants’ LCS exposure was associated with a higher risk of vomiting. However, supplemental analyses demonstrated that the increased risk was only observed among infants with LCS exposure of ≤1 serving/week. The presence of vomiting among infants with occasional, but not daily, LCS exposure may be explained by reverse causality. Mothers reporting infrequent DB intake may have previously been more frequent DB consumers but could have reduced their consumption after observing vomiting in their infants. However, given the mounting evidence demonstrating that maternal LCS ingestion during pregnancy and/or lactation may disturb the infants’ gut microbiota [11,12], the relationship between infant LCS exposure and vomiting requires further study. Notably, complete case analyses demonstrated that infant LCS exposure via maternal DB intake in four categories was not associated with increased risk of infants experiencing vomiting, suggesting the results are sensitive to the method used to address missingness in covariates.

In contrast to a recent study reporting that maternal DB intake during pregnancy is positively associated with infant body weight and risk of overweight in a population-based birth cohort of healthy pregnant women in Canada [19], no associations were observed between infants’ LCS exposure via maternal DB intake during lactation and infants’ weight or overweight status in the present analyses. However, our analyses were conducted on a smaller sample of infants exposed to the LCS in DBs over a relatively short time period, and captured maternal DB intake during lactation (postnatal DHQ) at a single time point, as opposed to in utero. Considering the growing body of preclinical evidence demonstrating adverse effects of early life LCS exposure both while in utero and via human milk on offspring’s weight and health [12,20], these null findings should be interpreted with caution. It is also possible that effects of LCS exposure on infant weight might not yet be apparent as early as three months of age, given that a prior study of early life LCS exposure reported associations with infant body weight at one year of age [19].

Strengths of our study include investigation of a nationally-distributed sample of lactating women in the US using data collected in the IFPSII, which allowed for comparisons across maternal sociodemographic and anthropometric characteristics. Our analysis was limited by the inability to assess exposure to specific LCS (e.g., sucralose, aspartame) and we were also unable to assess infants’ LCS exposure from maternal intake of LCS-containing foods, as this information was not captured by the postnatal DHQ administered in the IFPSII. Furthermore, no information on the timing of maternal LCS intake relative to the infants’ ingestion of human milk nor the consistency of maternal DB intake during the first three months postpartum was available, and therefore, our estimates were based on several assumptions. Assessment of infants’ health outcomes was also limited to weight, weight-for-age and BMI-for-age z-scores, overweight status, and mother-reported presence of gastrointestinal symptoms. In addition, rates of breastfeeding declined drastically in our sample after the three-month assessment, which precluded meaningful analyses of associations between maternal DB intake and infant outcomes at later timepoints.

## 5. Conclusions

Despite the lack of observed associations between infant exposure to LCS via maternal DB intake while breastfeeding and infant weight in this sample, there remains an urgent need to carefully investigate effects of infants’ LCS exposure via human milk on their future diet, weight, and health [8]. This information is critical to inform presently lacking recommendations for or against maternal DB consumption during lactation.

## Figures and Tables

**Table 1:** Maternal characteristics by reported postnatal diet beverage consumption (*n* = 682).

Reported Postnatal Diet Beverage Consumption ^1^				
	Never	≤1 Serving/Week	<1 Serving/Day	≥1 Serving/Day
**n (%) ^2^**	386 (56.6)	93 (13.6)	99 (14.5)	104 (15.3)
Age (years), mean (SD) **	29.3 (5.1)	29.9 (4.8)	30.6 (4.5)	31.7 (5.2)
Race/Ethnicity, *n* (%) **				
White	330 (55.8)	71 (12.0)	91 (15.4)	99 (16.8)
Black	12 (57.1)	6 (28.6)	1 (4.8)	2 (9.5)
Hispanic	20 (60.6)	6 (18.2)	6 (18.2)	1 (3.0)
Other	24 (64.9)	10 (27.0)	1 (2.7)	2 (5.4)
Household income, *n* (%) **				
<$35,000	134 (67.3)	16 (8.0)	24 (12.1)	25 (12.6)
$35,000–$74,999	182 (55.5)	48 (14.6)	47 (14.3)	51 (15.5)
≥$75,000	70 (45.2)	29 (18.7)	28 (18.1)	28 (18.1)
Education level, *n* (%) *				
High school or less	53 (67.1)	9 (11.4)	6 (7.6)	11 (13.9)
Some college	140 (59.6)	29 (12.3)	26 (11.1)	40 (17.0)
College or above	193 (52.4)	55 (14.9)	67 (18.2)	53 (14.4)
Marital status, *n* (%) **				
Yes	330 (54.5)	90 (14.9)	93 (15.4)	92 (15.2)
No	56 (72.7)	3 (3.9)	6 (7.8)	12 (15.6)
Gestational diabetes status, *n* (%) **				
Yes	17 (40.5)	5 (11.9)	6 (14.3)	14 (33.3)
No	369 (57.7)	88 (13.8)	93 (14.5)	90 (14.1)
Pre-pregnancy BMI (kg/m^2^), mean (SD) ***	25.1 (5.7)	25.3 (5.6)	26.9 (5.5)	28.3 (6.0)
Pre-pregnancy weight status, *n* (%) ^3,^***				
<25	229 (62.7)	59 (16.2)	44 (12.1)	33 (9.0)
25–29.9	82 (50.0)	16 (9.8)	31 (18.9)	35 (21.3)
≥30	70 (48.3)	18 (12.4)	23 (15.9)	34 (23.4)
**GWG (pounds), mean (SD)**	30.3 (12.5)	32.2 (12.9)	32.5 (14.2)	28.5 (15.3)
**Adherence to GWG recommendations, *n* (%) ^4^**				
Insufficient GWG	58 (56.3)	10 (9.7)	17 (16.5)	18 (17.5)
In healthy range	161 (61.2)	40 (15.2)	28 (10.6)	34 (12.9)
Excess GWG	167 (52.8)	43 (13.6)	54 (17.1)	52 (16.5)
Total energy intake (kcal/day), mean (SD) *	2002.4 (708.3)	1810.9 (648.0)	1875.6 (602.4)	1861.5 (592.2)
**Exclusive breastfeeding, *n* (%)**				
Yes	255 (58.6)	54 (12.4)	66 (15.2)	60 (13.8)
No	131 (53.0)	39 (15.8)	33 (13.4)	44 (17.8)

SD, standard deviation; BMI, body mass index; GWG, gestational weight gain. **^1^** Serving defined per medium serving specified on questionnaire (1 serving of diet soda = 12–16 ounces or 1 can or bottle; 1 serving of diet fruit drink = 8–16 ounces). Consumption categories presented are mutually exclusive. **^2^** Percentages may not add up to 100 due to rounding. **^3^** Pre-pregnancy weight status calculated based on standard adult CDC cut-offs for BMI. **^4^** Adherence to GWG recommendations was defined using GWG and pre-pregnancy BMI based on recommendations from Institute of Medicine. * *p* < 0.05; ** *p* < 0.01; *** *p* < 0.0001.

**Table 2:** The association between infants’ low-calorie sweetener exposure via maternal postnatal diet beverage intake and mean weight, weight-for-age z-score, and BMI-for-age z-score at 3 months of age.

	*n* ^1^	Exposed, Mean (SD)	Non-Exposed, Mean (SD)	Unadjusted Mean Difference (95% CI)	*p*-Value	Adjusted ^2^ Mean Difference (95% CI)	*p*-Value
Weight, kg	667	5.58 (0.83)	5.49 (0.89)	0.09 (−0.04, 0.23)	0.16	0.08 (−0.05, 0.21)	0.21
Weight-for-age z-score	664	−0.73 (1.14)	−0.81 (1.10)	0.07 (−0.10, 0.24)	0.40	0.03 (−0.14, 0.19)	0.76
BMI-for-age z-score	581	−0.33 (1.45)	−0.33 (1.33)	−0.01 (−0.23, 0.22)	0.96	−0.04 (−0.28, 0.20)	0.72

SD, standard deviation; CI, confidence interval. **^1^** Sample size for analysis of each infant outcome. **^2^** Adjusted for maternal age (years), race/ethnicity (white, black, Hispanic, other), household income (<$35,000, $35,000–$74,999, ≥$75,000), education level (high school or less, some college, college or above), marital status (yes/no) and gestational diabetes status (yes/no), pre-pregnancy BMI (kg/m^2^), gestational weight gain (GWG, pounds), total energy intake (kcal/day), sugar-sweetened beverage (SSB) intake (servings/month), infant’s birth weight (pounds), and exclusive breastfeeding (yes/no).

**Table 3:** The association between infants’ low-calorie sweetener exposure via maternal postnatal diet beverage intake and risk of any reported gastrointestinal (GI) symptoms and overweight at 3 months of age.

	*n* ^1^	Exposed *n* (%)	Non-Exposed *n* (%)	Unadjusted OR (95% CI)	*p*-Value	Adjusted ^2^ OR (95% CI)	*p*-Value
**Any reported GI symptoms, yes**	681	52 (17.6)	51 (13.2)	1.41 (0.92, 2.14)	0.11	1.38 (0.87, 2.20)	0.17
**Diarrhea, yes**	681	14 (4.7)	11 (2.8)	1.70 (0.76, 3.80)	0.19	1.54 (0.62, 3.79)	0.35
**Reflux, yes**	681	33 (11.2)	35 (9.1)	1.26 (0.76, 2.09)	0.36	1.09 (0.63, 1.90)	0.75
**Vomiting, yes**	681	14 (4.7)	9 (2.3)	2.09 (0.89, 4.89)	0.08	2.78 (1.05, 7.34)	0.04
**Overweight, yes ^3^**	575	26 (10.2)	33 (10.3)	0.99 (0.57, 1.70)	0.96	1.04 (0.57, 1.90)	0.91

OR, odds ratio; CI, confidence interval. ^1^ Sample size for each infant outcome analysis. ^2^ Adjusted for maternal age (years), race/ethnicity (white, black, Hispanic, other), household income (<$35,000, $35,000–$74,999, ≥$75,000), education level (high school or less, some college, college or above), marital status (yes/no) and gestational diabetes status (yes/no), pre-pregnancy BMI (kg/m^2^), gestational weight gain (GWG, pounds), total energy intake (kcal/day), sugar-sweetened beverage (SSB) intake (servings/month), infant’s birth weight (pounds), and exclusive breastfeeding (yes/no). ^3^ Infant overweight defined as weight-for-length z-score > 97.7th percentile, per WHO Child Growth Reference Standards.

## Data Availability

Publicly available datasets were analyzed in this study. This data can be found here: https://www.cdc.gov/breastfeeding/data/ifps/results.htm (accessed on 20 March 2021), and raw data can be requested by email to: ifps@cdc.gov.

## Supplementary Materials

The following are available online at https://www.mdpi.com/article/10.3390/nu13093154/s1, Table S1: The association between infants’ low-calorie sweetener exposure via maternal postnatal diet beverage intake and mean weight, weight-for-age z-score, and BMI-for-age z-score at 3 months of age, across four exposure categories ^1^, Table S2: Associations between infants’ low-calorie sweetener exposure via maternal postnatal diet beverage intake and risk of any reported gastrointestinal (GI) symptoms and infant overweight at 3 months of age, across four exposure categories^1^, Table S3: Associations between infants’ low-calorie sweetener exposure via maternal postnatal diet beverage intake and risk of vomiting at 3 months of age, complete case analysis (*n* = 585) ^1^.
