# Supplementary material for: Diet Beverage Intake during Lactation and Associations with Infant Outcomes in the Infant Feeding Practices Study II
Source: Nutrients. 2021 Sep 10;13(9):3154. doi: 10.3390/nu13093154 (PMC8472746; doi:10.3390/nu13093154)
Supplement: Supplementary file 1 [file nutrients-13-03154-s001.zip › nutrients-1342692-supplementary.pdf]

Supplement Table S1. The association between infants' low-calorie sweetener exposure via maternal postnatal diet beverage intake and mean weight, weight-for-age z-score, and weight-for-length z-score at 3 months of age, across four exposure categories[1]

	Never	≤1 serving/week	p-value[2]	<1 serving/day	p-value[2]	≥1 serving/day	p-value[2]
**Weight, kg**							
n[3]	377	98		103		89	
Mean (SD)	5.49 (0.89)	5.56 (0.83)	0.47	5.63 (0.82)	0.14	5.55 (0.84)	0.53
Adjusted[4] mean difference (95% CI)	Ref.	0.09 (-0.10, 0.27)	0.35	0.08 (-0.10, 0.26)	0.38	0.08 (-0.11, 0.27)	0.42
**Weight-for-age z-score**							
n[3]	374	98		103		89	
Mean (SD)	-0.81 (1.10)	-0.77 (1.14)	0.77	-0.64 (1.17)	0.18	-0.80 (1.12)	0.97
Adjusted[4] mean difference (95% CI)	Ref.	0.04 (-0.20, 0.27)	0.76	0.05 (-0.18, 0.28)	0.65	-0.02 (-0.26, 0.22)	0.88
**BMI-for-age z-score**							
n[3]	323	86		92		80	
Mean (SD)	0.33 (1.33)	-0.30 (1.58)	0.87	-0.40 (1.41)	0.67	-0.29 (1.34)	0.84
Adjusted[4] mean difference (95% CI)	Ref.	0.01 (-0.33, 0.35)	0.96	-0.17 (-0.50, 0.16)	0.31	0.04 (-0.30, 0.39)	0.80

SD, standard deviation; CI, confidence interval.

[1]Serving defined per medium serving specified on questionnaire (1 serving of diet soda= 12-16 ounces or 1 can or bottle; 1 serving of diet fruit drink= 8-16 ounces). Exposure categories presented are mutually exclusive.

[2]p-value for the comparison with the Never category

[3]Total sample size for each infant outcome analysis: weight, n = 667; weight-for-age z-score, n = 664; BMI-for-age z-score, n = 581.

[4]Adjusted for maternal age (years), race/ethnicity (white, black, Hispanic, other), household income (<$35,000, $35,000-$74,999, ≥$75,000), education level (high school or less, some college, college or above), marital status (yes/no) and gestational diabetes status (yes/no), pre-pregnancy BMI (kg/m^2), gestational weight gain (GWG, pounds), total energy intake (kcal/day), sugar-sweetened beverage (SSB) intake (servings/month), infant's birth weight (pounds), and exclusive breastfeeding (yes/no).

Supplement Table S2. Associations between infants' low-calorie sweetener exposure via maternal postnatal diet beverage intake and risk of any reported gastrointestinal (GI) symptoms and infant overweight at 3 months of age, across four exposure categories[1]

	Exposure	n	Prevalence, n (%)	Unadjusted OR (95% CI)	p-value	Adjusted[2] OR (95% CI)	p-value	p-value trend
Any reported GI symptoms (n=681)	Never	386	51 (13.2)	Ref		Ref		0.56
	≤1 serving/week	97	19 (19.6)	1.60 (0.89, 2.86)	0.11	1.72 (0.92, 3.21)	0.09	
	<1 serving/day	108	19 (17.6)	1.40 (0.79, 2.50)	0.25	1.39 (0.75, 2.58)	0.30	
	≥1 serving/day	90	14 (15.6)	1.21 (0.64, 2.30)	0.56	1.06 (0.54, 2.11)	0.86	
Diarrhea (n=681)	Never	386	11 (2.8)	Ref		Ref		0.22
	≤1 serving/week	97	3 (3.1)	1.09 (0.30, 3.98)	0.90	0.99 (0.25, 3.92)	0.98	
	<1 serving/day	108	6 (5.6)	2.01 (0.72, 5.55)	0.18	1.81 (0.59, 5.59)	0.30	
	≥1 serving/day	90	5 (5.6)	2.01 (0.68, 5.92)	0.21	1.86 (0.57, 6.04)	0.30	
Reflux (n=681)	Never	386	35 (9.1)	Ref		Ref		0.51
	≤1 serving/week	97	13 (13.4)	1.55 (0.79, 3.06)	0.20	1.55 (0.75, 3.23)	0.24	
	<1 serving/day	108	13 (12.0)	1.37 (0.70, 2.70)	0.36	1.23 (0.59, 2.53)	0.58	
	≥1 serving/day	90	7 (7.8)	0.85 (0.36, 1.97)	0.70	0.59 (0.24, 1.47)	0.26	
Vomiting (n=681)	Never	386	9 (2.3)	Ref		Ref		0.12
	≤1 serving/week	97	5 (5.2)	2.28 (0.75, 6.95)	0.15	3.49 (1.00, 12.1)	0.05	
	<1 serving/day	108	5 (4.6)	2.03 (0.67, 6.20)	0.21	2.56 (0.74, 8.90)	0.14	
	≥1 serving/day	90	4 (4.4)	1.95 (0.59, 6.47)	0.28	2.41 (0.65, 8.85)	0.19	
Infant overweight[3] (n=575)	Never	320	33 (10.3)	Ref		Ref		0.77
	≤1 serving/week	84	9 (10.7)	1.04 (0.48, 2.28)	0.91	1.04 (0.46, 2.39)	0.92	
	<1 serving/day	92	8 (8.7)	0.83 (0.37, 1.86)	0.65	0.83 (0.35, 2.00)	0.68	
	≥1 serving/day	79	9 (11.4)	1.12 (0.51, 2.44)	0.78	1.28 (0.55, 2.97)	0.56	

OR, odds ratio; CI, confidence interval.

[1]Serving defined per medium serving specified on questionnaire (1 serving of diet soda= 12-16 ounces or 1 can or bottle; 1 serving of diet fruit drink= 8-16 ounces). Exposure categories presented are mutually exclusive.

[2]Adjusted for maternal age (years), race/ethnicity (white, black, Hispanic, other), household income (<$35,000, $35,000-$74,999, ≥$75,000), education level (high school or less, some college, college or above), marital status (yes/no) and gestational diabetes status (yes/no), pre-pregnancy BMI (kg/m^2), gestational weight gain (GWG, pounds), total energy intake (kcal/day), sugar-sweetened beverage (SSB) intake (servings/month), infant's birth weight (pounds), and exclusive breastfeeding (yes/no).

[3]Infant overweight defined as weight-for-length z-score > 97.7th percentile, per WHO child growth reference standards.

Supplement Table S3. Associations between infants' low-calorie sweetener exposure via maternal postnatal diet beverage intake and risk of vomiting at 3 months of age, complete case analysis (n=585)[1]

Exposure	n	Prevalence n (%)	Unadjusted OR (95% CI)	p-value	Adjusted[2] OR (95% CI)	p-value	p-value trend
**2-category**							
Non-exposed	332	8 (2.4)	Ref		Ref		
Exposed	253	13 (5.1)	2.19 (0.90, 5.38)	0.08	3.20 (1.12, 9.13)	0.03	
**4-category[3]**							
Never	332	8 (2.4)	Ref		Ref		0.06
≤1 serving/week	85	4 (4.7)	2.00 (0.59, 6.81)	0.27	3.24 (0.81, 13.0)	0.10	
<1 serving/day	90	5 (5.6)	2.38 (0.76, 7.47)	0.14	3.47 (0.93, 13.0)	0.06	
≥1 serving/day	78	4 (5.1)	2.19 (0.64, 7.46)	0.21	2.93 (0.75, 11.4)	0.12	

OR, odds ratio; CI, confidence interval.

[1]Mother-infant dyads with missing data were excluded from this analysis.

[2]Adjusted for maternal age (years), race/ethnicity (white, black, Hispanic, other), household income (<$35,000, $35,000-$74,999, ≥$75,000), education level (high school or less, some college, college or above), marital status (yes/no) and gestational diabetes status (yes/no), pre-pregnancy BMI (kg/m^2), gestational weight gain (GWG, pounds), total energy intake (kcal/day), sugar-sweetened beverage (SSB) intake (servings/month), infant's birth weight (pounds), and exclusive breastfeeding (yes/no).

[3]Serving defined per medium serving specified on questionnaire (1 serving of diet soda= 12-16 ounces or 1 can or bottle; 1 serving of diet fruit drink= 8-16 ounces). Exposure categories presented are mutually exclusive.
